# Differences in lipid metabolism in acquired versus preexisting glucose intolerance during gestation: role of free fatty acids and sphingosine-1-phosphate

**DOI:** 10.1186/s12944-022-01706-x

**Published:** 2022-10-08

**Authors:** Moritz Liebmann, Katharina Grupe, Melissa Asuaje Pfeifer, Ingo Rustenbeck, Stephan Scherneck

**Affiliations:** Institute of Pharmacology, Toxicology and Clinical Pharmacy, Technische Universität Braunschweig, D-38106 Braunschweig, Germany

**Keywords:** Gestational diabetes mellitus (GDM), Prediabetes, Subpopulations, Heterogeneity of GDM, Sphingolipids, Sphingosine-1-phosphate, Sphingomyelin, CD36, PPARα, Pregnancy

## Abstract

**Background:**

The prevalence of gestational diabetes mellitus (GDM) is increasing worldwide. There is increasing evidence that GDM is a heterogeneous disease with different subtypes. An important question in this context is whether impaired glucose tolerance (IGT), which is a typical feature of the disease, may already be present before pregnancy and manifestation of the disease. The latter type resembles in its clinical manifestation prediabetes that has not yet manifested as type 2 diabetes (T2DM). Altered lipid metabolism plays a crucial role in the disorder's pathophysiology. The aim was to investigate the role of lipids which are relevant in diabetes-like phenotypes in these both models with different time of initial onset of IGT.

**Methods:**

Two rodent models reflecting different characteristics of human GDM were used to characterize changes in lipid metabolism occurring during gestation. Since the New Zealand obese (NZO)-mice already exhibit IGT before and during gestation, they served as a subtype model for GDM with preexisting IGT (preIGT) and were compared with C57BL/6 N mice with transient IGT acquired during gestation (aqIGT). While the latter model does not develop manifest diabetes even under metabolic stress conditions, the NZO mouse is prone to severe disease progression later in life. Metabolically healthy Naval Medical Research Institute (NMRI) mice served as controls.

**Results:**

In contrast to the aqIGT model, preIGT mice showed hyperlipidemia during gestation with elevated free fatty acids (FFA), triglycerides (TG), and increased atherogenic index. Interestingly, sphingomyelin (SM) concentrations in the liver decreased during gestation concomitantly with an increase in the sphingosine-1-phosphate (S1P) concentration in plasma. Further, preIGT mice showed impaired hepatic weight adjustment and alterations in hepatic FFA metabolism during gestation. This was accompanied by decreased expression of peroxisome proliferator-activated receptor alpha (PPARα) and lack of translocation of fatty acid translocase (FAT/CD36) to the hepatocellular plasma membrane.

**Conclusion:**

The preIGT model showed impaired lipid metabolism both in plasma and liver, as well as features of insulin resistance consistent with increased S1P concentrations, and in these characteristics, the preIGT model differs from the common GDM subtype with aqIGT. Thus, concomitantly elevated plasma FFA and S1P concentrations, in addition to general shifts in sphingolipid fractions, could be an interesting signal that the metabolic disorder existed before gestation and that future pregnancies require more intensive monitoring to avoid complications.

**Graphical Abstract:**

This graphical abstract was created with BioRender.com.
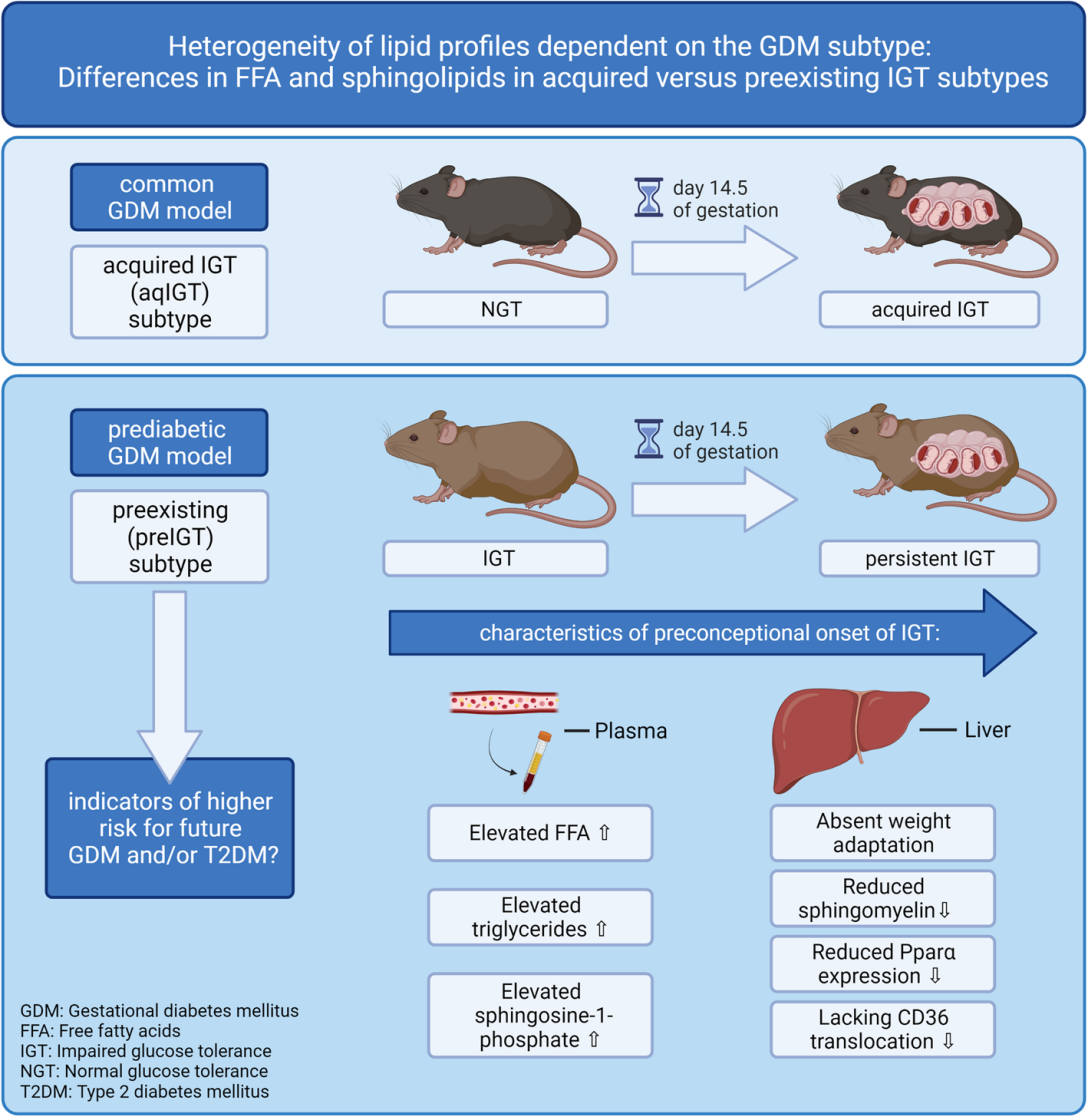

## Introduction

Gestational diabetes mellitus (GDM) is a complex metabolic disease that is diagnosed for the first time during pregnancy. The International Diabetes Federation (IDF) determined an overall global prevalence of GDM of 14.2% in 2021 [1]. It has become one of the most serious diseases during pregnancy, associated with severe consequences such as diabetes and obesity for the child and a higher risk of complications for the mother. The main characteristic according to the clinical definition of GDM is impaired glucose tolerance (IGT) rather than persistent hyperglycemia, as in T2DM [2]. An essential component of the pathogenesis is the inadequate ability of pancreatic β-cells to compensate for the physiological insulin resistance (IR) during pregnancy. However, the severity of the disease and additional comorbidities can differ considerably. For this reason and to select optimal treatment strategies, there is evidence to classify GDM into subcategories, as in T2DM. Factors such as obesity, IR, fasting blood glucose, the diagnostic results of an oral glucose tolerance test (OGTT), but also dyslipidemia play an important role in this context. In this regard, free fatty acids (FFA) and other plasma lipids can be used as markers, but they may also play a functional role in the development of GDM. However, it should be considered that the expectant mother develops physiological IR to ensure nutrient supply to the fetus [3–5]. This physiological condition is also associated with increased plasma lipids, such as total cholesterol (TC), triglycerides (TG), and cholesterol in low-density lipoprotein (LDL-C) as well as very-low-density lipoprotein (VLDL-C) which rise progressively [6–8].

Little is usually known about the glucose and lipid metabolism of individual patients prior to pregnancy. The reason for this is that the OGTT used for diagnosis is usually performed for the first time during pregnancy. Tests without clinical indicators of disease are not usually performed before conception. Thus, if GDM is diagnosed for the first time during pregnancy, there are two possible conclusions: either the disease is a new onset during pregnancy or the impaired glucose tolerance was already present asymptomatically before pregnancy. Harris addressed as early as 1988 that GDM and/or gestational impaired glucose tolerance (g-IGT) may also be a preconception disorder diagnosed first during pregnancy [9, 10]. This can therefore be described as a preexisting IGT and should not be confounded with manifest diabetes, which is first diagnosed during pregnancy. If disease severity and course of GDM and preconceptional IGT differ during pregnancy, differential diagnosis of these GDM subtypes could lead to better monitoring of patients. This is of importance because a large proportion of patients with a confirmed diagnosis of GDM have a recurrence risk of the disease in a subsequent pregnancy. They also have an increased risk of developing T2DM later in life [11]. From our point of view, it is obvious that patients with preconceptional glucose intolerance and in the presence of other risk factors belong to this high-risk population. However, very few data are available to stratify increased disease risk from the presence of these factors.

**Fig. 1:**
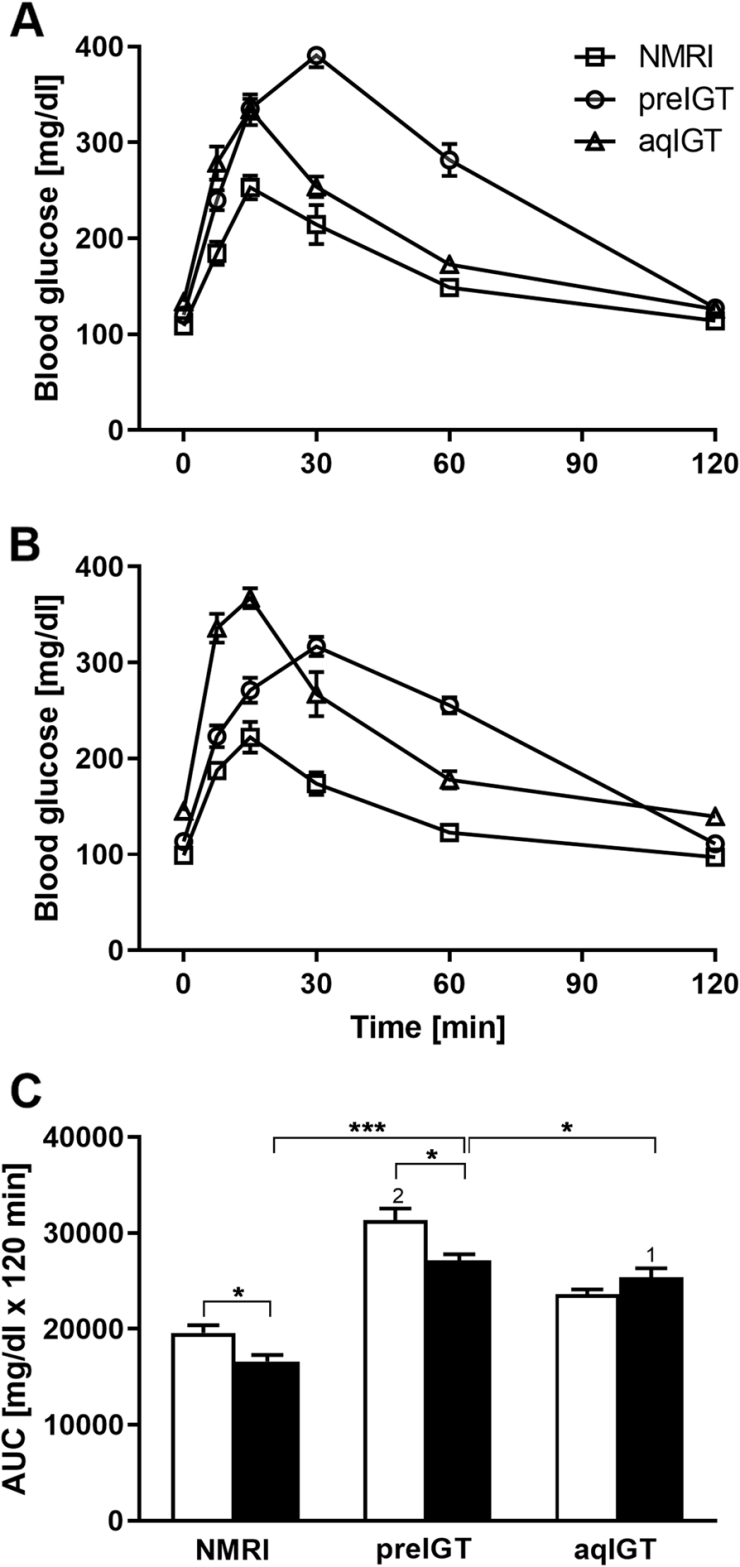
Differences in the manifestation of impaired glucose tolerance in preIGT and aqIGT mice. Blood glucose concentrations during 120 min of oral glucose tolerance tests (OGTT) of mice with preexisting impaired glucose tolerance (preIGT; circles), impaired glucose tolerance acquired during gestation (aqIGT; triangles), and NMRI mice (squares), at time points **A** preconceptional (pc.) and **B** day 14.5 of gestation(d14.5). **C** Area under the curve (AUC) for blood glucose calculated by the trapezoidal rule of preIGT, aqIGT, and NMRI mice at time points pc. (white bars) and d14.5 (black bars). Data are presented as means ± SEM (*n* = 7–11 animals per group). *p < 0.05, ***p < 0.001, NMRI pc. vs. aqIGT pc.: 1*p* < 0.05, NMRI pc. vs. preIGT pc.: 2*p* < 0.0001. Part of the data has already been published (NMRI and preIGT datasets over a period of 0–120 min) in Grupe et al., 2020

There are a variety of different rodent models for the study of GDM. These differ with regard to the initiation of the phenotype and vary between surgical, pharmacologically-induced, diet-induced, and genetically manipulated models [12]. The New Zealand obese (NZO) mouse is a polygenic model with a long history in diabetes research. It is well established in the fields of obesity and T2DM. Males are characterized by hyperglycemia, hyperinsulinemia, β-cell failure, and hypoinsulinemia in later life [13–16]. In contrast, female mice develop only IGT. However, this can become overt diabetes under metabolic stress conditions e.g. following the feeding of a high-fat diet (HFD) or after ovariectomy [17, 18]. In a recent study, we investigated glucose metabolism and adaptation of islets of Langerhans in NZO mice during gestation. The animals were compared with the metabolically intact NMRI mouse as a control. We demonstrated that the preconceptionally (pc.) IGT persists during gestation. Interestingly, IGT did not deteriorate but showed a slight improvement [19]. Thus, the NZO mouse should be considered as a model with IGT before and during gestation. In contrast, common GDM shows only a transient glucose tolerance impairment during gestation. C57BL/6 mice are among the most commonly used strains in animal research. C57BL/6 substrains are well-established models for diet-induced obesity, IGT, and therefore IR. The mice have also been considered as a model for GDM, but only after treatment with a HFD [20, 21]. To obtain a model with common GDM progression even on a standard diet, which is then comparable to the NZO strain in the experimental conditions, we examined the substrain C57BL/6 N. We first performed OGTTs and simultaneously determined glucose-stimulated insulin secretion (GSIS). In contrast to NZO, the C57BL/6 N strain showed IGT only during gestation (Fig. 1).

**Fig. 2:**
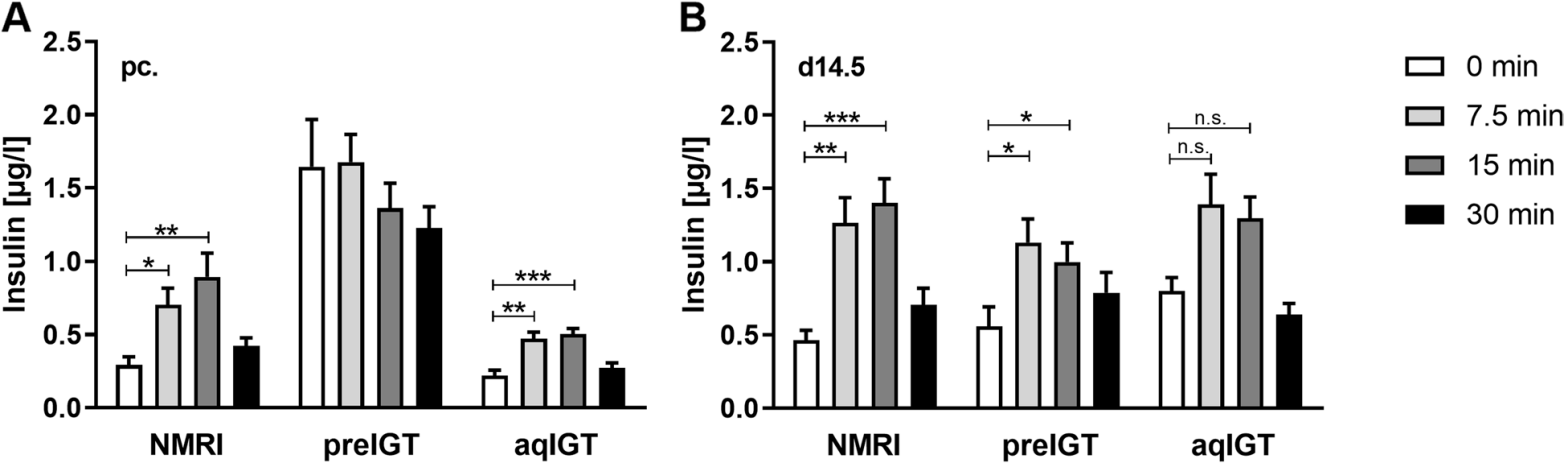
Improved stimulability of insulin secretion in preIGT and deterioration in aqIGT mice during gestation. Plasma insulin during the first 30 min of oral glucose tolerance test (OGTT) of mice with preexisting impaired glucose tolerance (preIGT), impaired glucose tolerance acquired during gestation (aqIGT), and NMRI mice, at time points **A** preconceptional (pc.) and **B** day 14.5 of gestation (d14.5). Data are presented as means ± SEM (*n* = 8–11 animals per group). *p < 0.05, **p < 0.01, ***p < 0.001. Part of the data has already been published (NMRI and preIGT datasets) in Grupe et al., 2020

The strains showed important differences in the GSIS profile (Fig. 2). Preconceptionally, the NZO mouse shows hyperinsulinemia characterized by insufficient stimulability of insulin secretion by glucose. Stimulability is present in both C57BL/6 N and NMRI, with the latter strain characterized by slightly higher insulin concentrations after oral glucose application. During gestation, insulin secretion remains stimulable in the NMRI control. The NZO strain also showed a significant improvement in stimulability in contrast to the preconceptional state. In C57BL/6 N, a trend toward deterioration in stimulability of insulin secretion by glucose was observed, independent of absolutely elevated insulin concentrations (Fig. 2B). After weaning, all three strains returned to the preweaning state of secretion, albeit to different degrees [19].

These data indicate differences between the NZO and C57BL/6 N phenotypes, which can be divided into IGT already present preconceptionally and IGT acquired during gestation exactly analogous to what Harris addressed before [10]. Therefore, we distinguish between a preexisting IGT phenotype (preIGT, NZO) and an IGT phenotype acquired during gestation (aqIGT, C57BL/6 N) compared with the healthy NMRI control strain.

Suitable biomarkers for GDM and the progression of a preconceptional prediabetic phenotype are nevertheless sparse. For this reason, various potential markers, such as low adiponectin and sex hormone binding globulin (SHBG) levels, have already been examined for suitability for the prediction of GDM [22–24]. Of particular interest are predictive markers for the identification of prediabetes, with already impaired IGT or impaired fasting glucose (IFG), which may precede manifest diabetes [25]. Studies suggest that IGT-specific metabolites of lipid metabolism are indicative in prediabetic cohorts [24]. Other characteristic changes in the lipid fractions, such as hyperlipidemia, with increased FFA concentrations in the plasma, as well as changes in the sphingolipid fraction have been described [26, 27]. Lipidomics analysis in GDM cohorts identified TG as suitable biomarkers in serum samples in the early second trimester [28]. Lai et al. also identified TG and sphingolipids such as sphingomyelin (SM) as markers for the progression of GDM to T2DM [29]. Further increased spjhingosine-1-phosphate (S1P) concentrations in plasma are linked to human and rodent obesity [30]. Hence, the inhibition of insulin signaling by S1P over the S1P2 receptor was observed as well as the increase of extra- and intracellular S1P in primary rat hepatocytes after palmitate stimulation [31]. These data indicated a potential increase in insulin resistance mediated by S1P. There is evidence that differences in the onset of IGT manifestation and thus also in the pathophysiology GDM subtypes exists and the relationship between lipid metabolism and GDM has become stronger recently. In the prospective cohort from the UPBEAT trial GDM in obese patients was linked to elevated plasma diglycerides, TG and FFA concentrations, consistent with de novo lipogenesis [32]. These alterations could even be associated with neonatal abdominal circumference. A further study observed more pronounced increases in TG, as well as VLDL and LDL lipoproteins during pregnancy in obese women compared to lean controls [33]. Other data did not show any differences in plasma cholesterol, HDL-C, LDL-C and TG between GDM patients and healthy controls, but decreased HDL-C and elevated concentrations of the other lipid classes three month after child birth [34]. Gueuvoghlanian-Silva et al. were able to distinguish between severe, insulin-treated, and milder forms of GDM depending on serum lipid profiling [35]. The dyslipidemic state during pregnancy was more intense in severe GDM compared to a milder form or even healthy pregnant women. Even differences between breast-fed and formula-fed infants could be found in the lipid profile and for the latter an increased infant weight could be associated with increased levels in the SM lipid class at 3 months of age [36]. However, the data are contradictory. In some studies, GDM is associated with significantly increased lipid concentrations in all trimesters, while in others no increasing lipid concentrations were observed compared to metabolically healthy controls [37, 38]. Further, to our knowledge, there is a lack of data on GDM subtypes with preconceptional onset of IGT, as many studies lack a preconceptional time point, even just to ensure that changes due to pregnancy-related effects e.g. hormonal changes can excluded.

We therefore developed the hypothesis that the different initial onset of IGT is indicated by changes in lipid constitution. This study aimed to investigate whether differences in lipid metabolism exist depending on the time of onset of glucose tolerance impairment (preIGT vs. aqIGT). Therefore, the major plasma lipid classes and especially the lipid metabolism of the liver of the mouse models should be compared at the time points preconceptional and during gestation.

## Material and methods

### Animals

All procedures were approved by the ethics committee of the Lower Saxony State Office for Consumer Protection and Food Safety (Oldenburg, Germany; ethics approval number: 33.19–42,502-04–17/2462; internal IDs (05.15) TSB TU BS and (05.19) TSB TU BS). The NZO (NZO/HIBomDife), B6 (C57BL/6NCrl), and the NMRI (NMRI/RjHan) strains were used for this study. The NMRI outbred strain is an established control in obesity and diabetes research [39]. A significant advantage of this outbred strain is the genetic heterogeneity within the population, which is analogous to humans and thus leads to improved transferability from animal models to humans. Unlike inbred strains, the outbred strain does not react singularly to environmental influences or experimental interventions. Due to several genetic variants, with incomplete to complete penetrance, the outbred strain has the entire allelic spectrum in each biological signalling pathway, so that it can respond physiologically adequately to manipulations [40, 41]. Female NMRI mice exhibit increased body weight on standard diets and are thus well suited as a comparison group for the obese NZO strain. Moreover, as a metabolically healthy control, the NMRI strain exhibits a normal physiological adaptation to pregnancy and robust β-cell physiology in the presence of a physiological stressor with an increased litter size [19, 42]. Furthermore, NMRI do not show significant metabolic changes or even manifest diabetes mellitus even on HFD [43].Thus, metabolically healthy female NMRI mice are suitable as controls in all comparison groups of this work. [19]. Mice were housed in an air-conditioned room at 21 ± 1 °C with light/dark periods of 12:12 h (lights on at 06:30 am). Animals had ad libitum access to water and food and were fed a standard chow diet (SD; 1328 P, Altromin, Lage, Germany) containing 11% fat, 24% protein, and 65% carbohydrates with a total metabolizable energy of 13.5 kJ/g. Female NZO and NMRI mice were mated overnight at the age of about 7 weeks. Due to smaller body size and weight, matings of the B6 strain were scheduled at 8–10 weeks of age. Pregnancy was indicated successful by the presence of copulatory plugs the following morning. This day was denoted as 0.5 days *post coitum* and mice were studied on day 14.5 of gestation (d14.5) at the age of about 9–10 weeks, fed ad libitum. The preconceptionally (pc.) examined group was the same age as the pregnant one. These animals were used for all performed tissue and plasma-related experiments as well as histological analysis. The same animals were studied in vivo over the period indicated below to determine changes in glucose tolerance.

### Oral glucose tolerance test (OGTT)

After six hours of food deprivation, basal blood glucose and insulin plasma concentrations of NZO, B6 and NMRI mice were determined. At time points 7.5, 15, 30, 60, and 120 min after application, blood glucose and at 7.5, 15, and 30 min insulin concentrations were measured from the tail tip. The experimental conditions have already been described [19, 44]. The NZO mice and NMRI controls blood glucose and insulin concentration datasets have been previously published [19]. These data have now been extended to include the B6 strain and are presented together with the new data for better illustration.

### Tissue collection

Mice were sacrificed by cervical dislocation. Before dissection, total body weight, maternal liver, and fetal weight were obtained to determine weight gain in response to pregnancy. Maternal livers were dissected and snap-frozen in liquid nitrogen. Tissue samples were pestled to powder, apportioned, and stored at − 80 °C until further use. For histological staining tissue samples were fixed in 4% neutral phosphate-buffered formaldehyde for 24 h. Fixed tissues were then embedded in paraffin according to standard procedures [45]. Following fixation, serial sections of 4 μm were prepared for immunocytochemistry.

### Plasma lipid and lipoprotein surrogate analysis

Whole blood was collected via the *vena cava* or the heart after isoflurane anesthesia followed by cervical dislocation. Plasma was obtained by centrifugation at 21.000 g for 2 min at 4 °C and samples were stored at − 80 °C until further use. Plasma samples were analyzed by colorimetric and fluorometric assays according to the manufacturer’s protocols for concentrations of FFA (Sigma, Steinheim, Germany), triglycerides (TG; Sigma, Steinheim, Germany), cholesterol (CL; Abcam, Cambridge, UK), surrogate values HDL-C and LDL/VLDL-C after separation of HDL and LDL/VLDL by precipitation and centrifugation(Abcam, Cambridge, UK), SM (Cell Biolabs, San Diego, USA), and S1P (Abbexa, Cambridge, UK). Plasma S1P was measured with a commercially available competitive ELISA. The S1P ELISA measures total S1P concentrations in plasma without distinction between lipoprotein/albumin bound S1P fractions [46].

### Hepatic lipid analysis

TG, CL, and SM standards were purchased from Alfa Aesar (Haverhill, USA), Merck (Darmstadt, Germany), and Sigma (Steinheim, Germany). 100 mg of the snap-frozen hepatic samples were thawed before extraction. 1000 µg of oleyl alcohol (Sigma, Steinheim, Germany) was added as an internal standard. The tissue was dispersed with a mixture of chloroform–methanol (2:1, v/v) and homogenized with an ultrasonic homogenizer under ice-cooling. After filtering, the homogenates were extracted with chloroform–methanol (2:1, v/v) according to the Folch procedure [47]. The extracts were evaporated to constant mass, dissolved in chloroform–methanol (1:1, v/v), and applied in equal amounts to a solid phase. Extraction of lipids was carried out using a NH_2_-aminopropylic disposable cartridge column (Macherey & Nagel, Düren, Germany) according to a standard procedure [48]. Each collected fraction was evaporated and dissolved in chloroform–methanol (1:1, v/v). Precoated TLC (thin layer chromatography) plates (silica gel 60 F254; Macherey & Nagel, Düren, Germany) were used as stationary phase. The plates were prewashed with hexane or chloroform–methanol (2:1, v/v). Afterwards, the plates were activated at 110 °C for 15 min and then cooled in a vacuum desiccator. The samples and standard substances were applied onto the plates with a semiautomatic Linomat 5 TLC sample dispenser (Camag, Muttenz, Switzerland), and the spotting volume was 1.0 µl. The lipids were chromatographed at 20 °C. The first solvent system contained chloroform–methanol-acetic acid (30:20:10, v/v/v) and was stopped when the solvent front ascended to 6 cm above the bottom edge. The plates were dried and subjected to chromatography in a second solvent system consisting of hexane-toluol-acetic acid (30:20:2, v/v/v), ascending to 12 cm above the bottom edge. Thereafter, the plates were dried and subjected to chromatography in a third solvent system consisting of hexane ascending to the top of the plate. Following the development, the plates were dried at 100 °C. The plates were sprayed with 10% (w/v) cupric sulfate in 8% (v/v) phosphoric acid solution and heated at 180 °C for 20 min [49]. Subsequently, the plates were scanned immediately by a CD60 HPTLC-densitometer (Desaga, Wiesloch, Germany) at a wavelength of 535 nm. Lipid fractions were identified by matching their Rf values and the integrated area under curve values, with analysis standards. FFA were confirmed in hepatic extracts obtained by the method of Bligh and Dyer by a fluorometric assay kits (Sigma, Steinheim, Germany) according to manufacturer's protocols [50]. To identify the sphingolipid class, SM was measured by a fluorometric assay (Cell Biolabs, San Diego, USA) and S1P by a competitive ELISA kit (Abbexa, Cambridge, UK) according to manufacturer's protocols and *Pei *et al*.* [51].

### Immunocytochemistry and histological assessment of hepatic sections

After rehydration of the formalin-fixed, paraffin-embedded hepatic tissue sections, immunocytochemistry was performed using rabbit polyclonal anti-fatty acid translocase (FAT/CD36) antibody (1:100; PA1-16,813 Thermo Fisher Scientific, Waltham, USA) and rabbit polyclonal anti- peroxisome proliferator-activated receptor alpha (PPARα) antibody (1:600; PA1-822A Thermo Fisher Scientific, Waltham, USA). After overnight incubation at 4 °C, primary antibodies were detected with fluorophore-labeled secondary antibody Alexa Fluor488 goat anti-rabbit (1:500; Jackson ImmunoResearch, West Grove, USA) including DAPI (1:1000; KPL, Gaithersburg, USA) nuclear counterstain in light-shielded conditions. Two sections per animal (*n* = 3–5 animals per group) were used for CD36 evaluation and three sections per animal (*n* = 3–5 animals per group) were used for PPARα evaluation. Acquisition of representative images was performed with the upright microscope Eclipse Ni-E (Nikon, Düsseldorf, Germany), equipped with the DS-Fi3 Color Camera (Nikon, Düsseldorf, Germany). For histological assessment, CD36 quantification was performed measuring integrated fluorescence density after Hammond with ImageJ (NIH, USA) and membrane fluorescence intensity with NIS elements AR 5 (Nikon, Düsseldorf, Germany) [52, 53]. PPARα integrated fluorescence density was analyzed after Jensen with ImageJ (NIH, USA) [54].

### Real-time PCR

RNA extraction and quantitative Real-time PCR were performed using previously described methods [55–57]. Real-time PCR was performed with an Applied Biosystems 7500 Fast Real-time PCR system (Thermo Fisher Scientific, Waltham, USA) and the QuantiNova SYBR® Green PCR Kit (Qiagen, Hilden, Germany). The expression of a target gene was normalized to the housekeeping genes Actin Beta *(Actb*), Hypoxanthine Phosphoribosyltransferase 1 (*Hprt1),* and Peptidylprolyl Isomerase A (*Ppia)*. The following primer sequences were constructed using the PrimerBank and Primer-BLAST databases for *Cd36* (NM_001159555) and *Pparα* (NM_001113418) real-time analysis (Table 1) [58, 59].

**Table 1:** Primers used in Real-time PCR analysis of *Cd36* and *Pparα* gene expression

Gene	Forward primer sequence	Reverse primer sequence
*Actb*	5’-ccc ctg aac cct aag gcc a-3’	5 ‘-cgg act cat cgt act cct gc-3 ‘
*Hprt1*	5’-cag tcc cag cgt cgt gat ta-3’	5’-ggc ctc cca tct cct tca tg-3’
*Ppia*	5’-cgt gtt ctt cga cat cac ggc-3’	5’-tgg cgt gta aag tca cca ccc-3’
*Cd36*	5’-tgt gtt tgg agg cat tct ca-3’	5’-ttt tgc acg tca aag atc ca-3’
*Pparα*	5’-tgc agc ctc agc caa gtt gaa-3’	5’-ttc ccg aac ttg acc agc ca-3’

### Statistical analysis

Statistical analysis and graphical presentation were performed by Prism 9 (GraphPad, La Jolla, USA). Data are presented as means ± SEM. Due to the sample size data were classified as non-parametric. To compare the means of two strains separately for each of the time points or to compare differences within a strain over the time period (e.g. NMRI pc. vs. NMRI d14.5), the Mann–Whitney U test was applied. To analyze alterations of more than two independent samples within two strains over the time period, a Kruskal–Wallis H test followed by a Dunn’s multiple-comparison test was applied. A value of *p* < 0.05 was considered statistically significant. *P* values were indicated as *p < 0.05, **p < 0.01, ***p < 0.001 and ****p < 0.0001.

## Results

### Differences in plasma lipid classes between preIGT and aqIGT mice

**Fig. 3:**
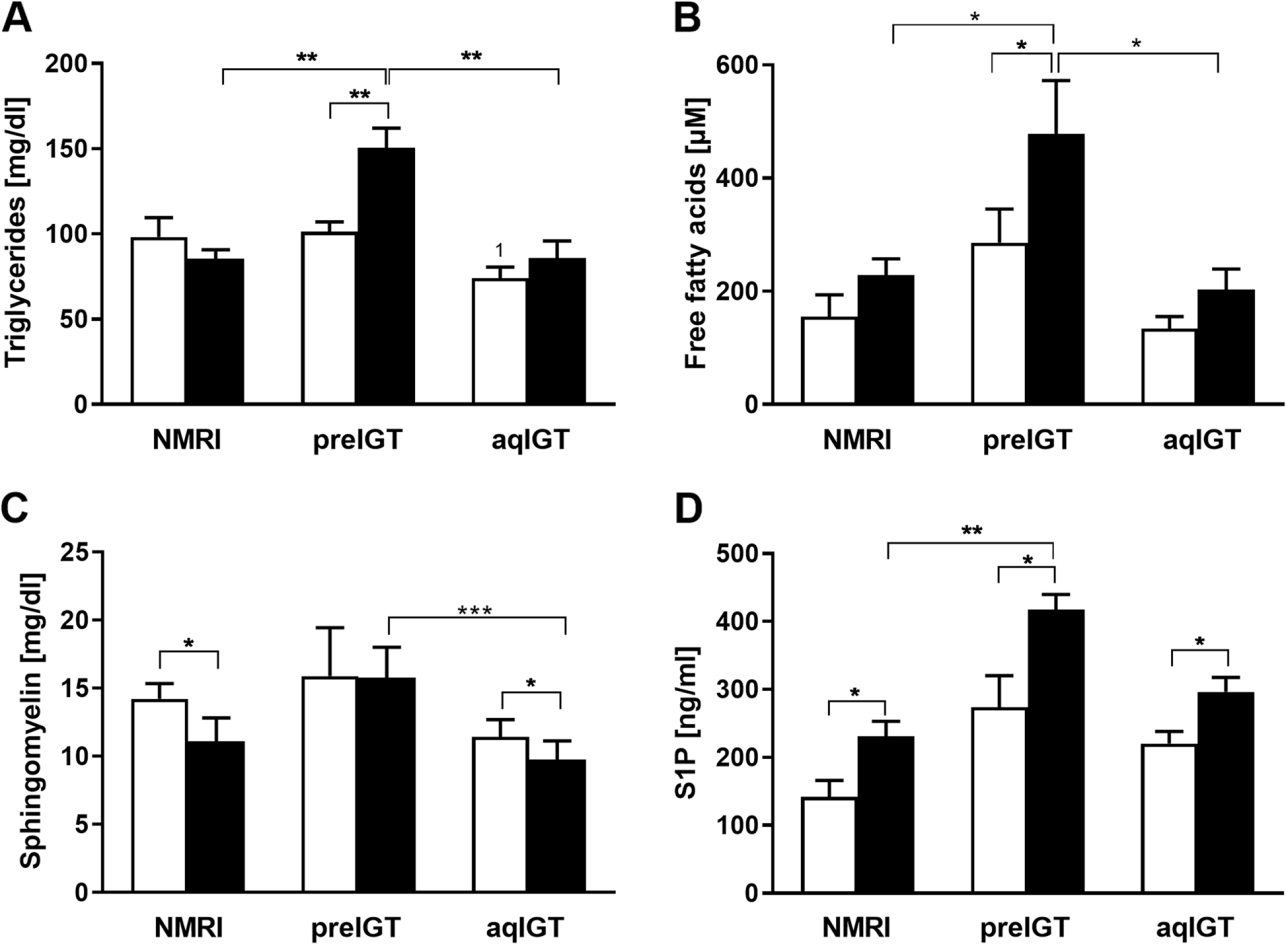
Hyperlipidemia in preIGT mice during gestation. **A** Concentrations of plasma triglycerides, **B** free fatty acids, **C** sphingomyelin, and **D** sphingosine-1-phosphate (S1P) of mice with preexisting impaired glucose tolerance (preIGT), impaired glucose tolerance acquired during gestation (aqIGT), and NMRI mice, at time points preconceptional (pc.; white bars) and day 14.5 of gestation (d14.5; black bars). Data are presented as means ± SEM (**A** *n* = 6–11, **B** *n* = 6–10, **C** *n* = 15, and **D** *n* = 5 animals per group). *p < 0.05, **p < 0.01, ***p < 0.001, preIGT pc. vs. aqIGT pc.: 1*p* < 0.05

To investigate changes in lipid metabolism, important plasma lipid fractions were first examined. Major plasma classes were measured in all three strains at both time points preconception and d14.5. Preconceptionally TG did not differ between preIGT and NMRI controls, while preIGT mice showed significantly higher concentrations than aqIGT mice (Fig. 3A). In both, NMRI and aqIGT strains, the TG concentrations did not vary between preconception and d14.5. However, there was a significant increase in preIGT TG concentrations at d14.5, displaying significantly higher plasma TG concentrations than the other two comparators, as well as hypertriglyceridemia (preIGT vs. aqIGT vs. NMRI: 150.41 ± 11.68 vs. 85.93 ± 10.06 vs. 85.56 ± 5.19 mg/dl; preIGT vs. aqIGT: *p* < 0.01, preIGT vs. NMRI: *p* < 0.01). The same proportions as in the TG fraction were found in the plasma FFA fraction. Preconceptionally, FFA did not differ between preIGT and NMRI controls, whereas preIGT mice showed significantly higher concentrations than aqIGT mice. During pregnancy, only the preIGT mice showed a significant increase compared to preconception. At d14.5, the plasma concentrations in the preIGT mice were twofold higher than in the latter two strains (preIGT vs. aqIGT. vs. NMRI: 477.93 ± 94.34 vs. 202.50 ± 36.81 vs.228.17 ± 29.17; preIGT vs. aqIGT: *p* < 0.05, preIGT vs. NMRI: *p* < 0.05), revealing further signs of dyslipidemia (Fig. 3B).

**Fig. 4:**
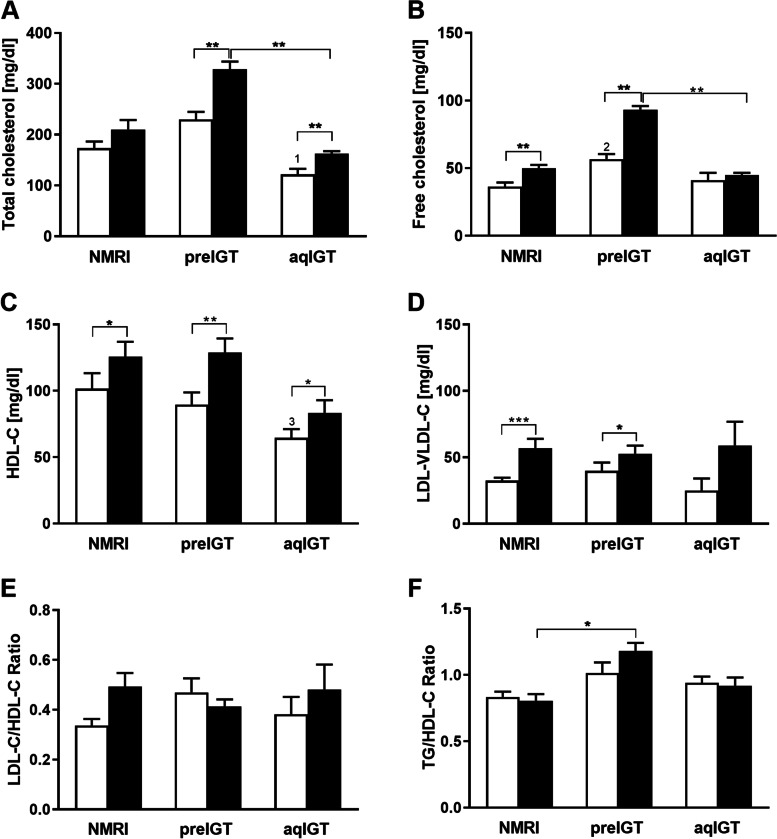
Hypercholesterolemia but lacking shift in the lipoprotein HDL-C/LDL-C ratio in preIGT mice during gestation. **A** total plasma cholesterol, **B** free cholesterol and the lipoprotein surrogate values **C** high density lipoprotein cholesterol (HDL-C) and **D** low/very low density lipoprotein cholesterol (LDL-VLDL-C) of mice with preexisting impaired glucose tolerance (preIGT), impaired glucose tolerance acquired during gestation (aqIGT), and NMRI mice, at time points preconceptional (pc.; white bars) and day 14.5 of gestation (d14.5; black bars). **E** Calculated LDL-VLDL-C to HDL-C-ratio and **F** atherogenic index of plasma (Triglyceride/HDL-C). Data are presented as means ± SEM (**A** and **B** *n* = 5–6, **C** and **D** *n* = 5 animals per group). *p < 0.05, **p < 0.01, ***p < 0.001. preIGT pc. vs. aqIGT pc.: 1*p* < 0.01; NMRI pc. vs. preIGT pc.:2*p* < 0.05; preIGT pc. vs. aqIGT pc.: 3*p* < 0.05

Since sphingolipids have essential roles in energy homeostasis and are linked to adverse metabolic conditions, this fraction was further analyzed. Preconceptionally, there were no differences in the SM fraction between all three observed strains. While both aqIGT and NMRI mice showed decreased concentrations at d14.5, the SM concentrations did not decline in preIGT mice (Fig. 3C). The S1P concentrations revealed a similar distribution pattern as the FFA fraction. Preconceptionally, there was no difference between all three strains. At d14.5, preIGT mice exhibited significantly increased S1P concentrations compared to NMRI controls (preIGT vs. NMRI: 417.49 ± 22.46 vs. 231.17 ± 21.95 ng/ml; *p* < 0.01) (Fig. 3D). Further, the CL fractions were examined. Preconceptionally, there was no difference between preIGT mice and NMRI controls in total cholesterol (TC), while preIGT mice showed significantly higher concentrations than aqIGT mice. The preIGT mice also revealed increased concentrations at d14.5, which were significantly elevated compared to aqIGT and over 1.5 times higher than the NMRI controls (preIGT vs. aqIGT vs. NMRI: 328.64 ± 15.08 vs. 162.58 ± 4.94 vs. 210.30 ± 18.54 mg/dl; preIGT vs. aqIGT: *p* < 0.01, preIGT vs. NMRI: *p* = 0.100) (Fig. 4A). Regarding the free cholesterol (FC) fraction, preconceptionally, the preIGT mice exhibited significantly higher concentrations than the NMRI controls. Following this pattern, the FC fraction in preIGT mice at d14.5 was also significantly higher than in the aqIGT model and also noticeably higher than in NMRI controls (Fig. 4B). Thus, the preIGT mice showed signs of hypercholesterolemia during gestation. To derive important laboratory diagnostic indices, plasma lipoproteins were separated to resolve the lipoprotein surrogate values LDL-cholesterol/HDL-cholesterol ratio (LDL-C/-HDL-C) as well as the plasma TG/HDL-cholesterol ratio (TG/HDL-C) and the atherogenic index (Fig. 4C-F). All three strains showed tendencies towards elevated HDL-C and LDL-C concentrations at d14.5, which were equally distributed, resulting in no significant shifts in the LDL-C/HDL-C ratio observed during pregnancy (Fig. 4E). The atherogenic index increased in NZO mice at d14.5 due to the higher plasma TG concentrations during gestation (Fig. 3A & Fig. 4E). Compared to agIGT and NMRI mice, preIGT mice showed hyperlipidemia at d14.5 characterized by increased plasma TG, FFA as well as TC concentrations.

### Lack of gestational weight adaptation of livers of preIGT mice

**Fig. 5:**
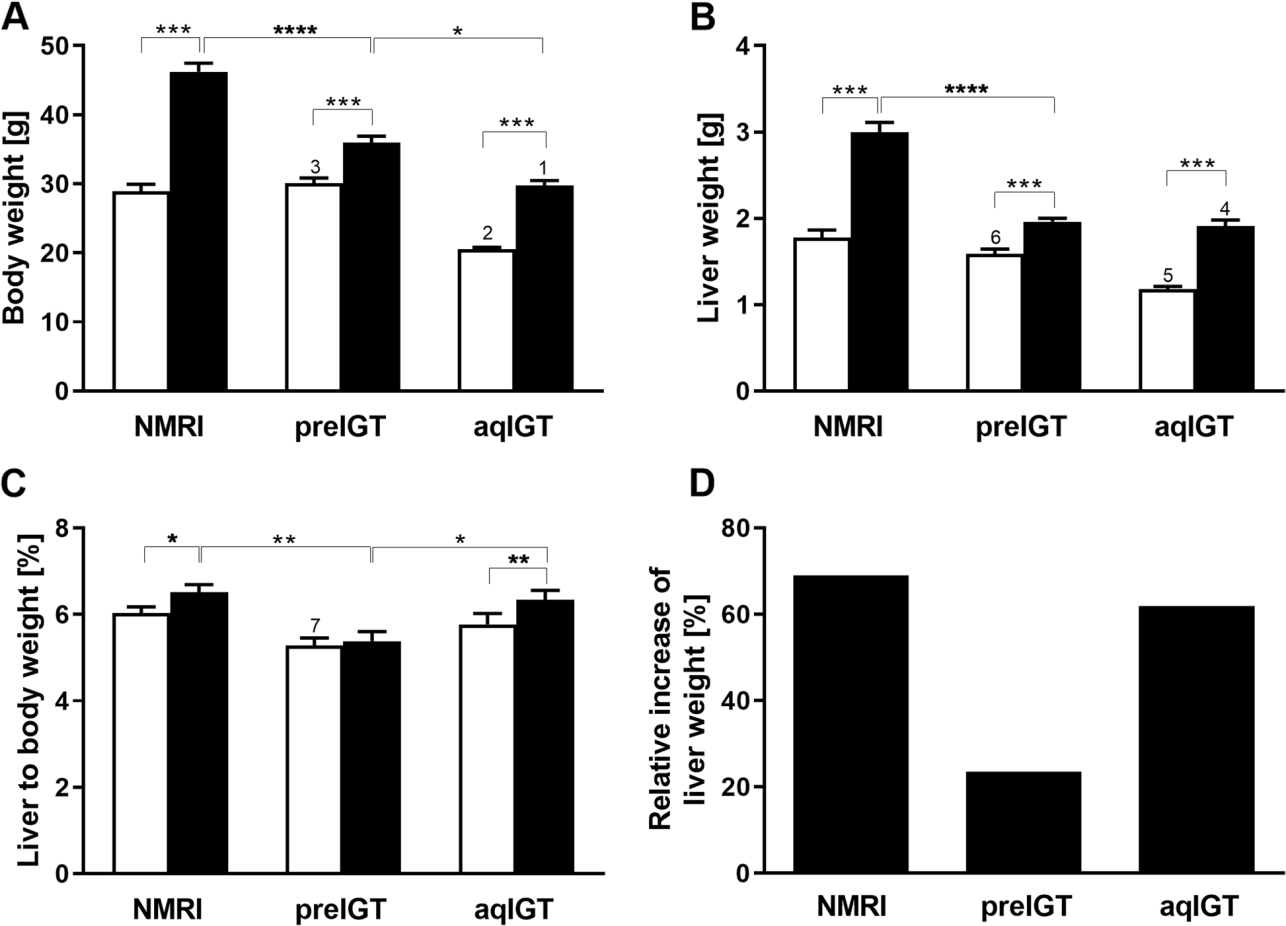
Differences in body and liver weight adjustment in gestation. **A** Body weight, **B** liver weight and **C** liver to body weight-ratio of mice with preexisting impaired glucose tolerance (preIGT), impaired glucose tolerance acquired during gestation (aqIGT), and NMRI mice, at time points preconceptional (pc.; white bars) and day 14.5 of gestation (d14.5; black bars). **D** Relative increase in liver weight at d14.5. Data are presented as means ± SEM (*n* = 11–14 animals per group). *p < 0.05, **p < 0.01, ***p < 0.001, ****p<0.0001, NMRI d14.5 vs. aqIGT d14.5: 1*p* < 0.0001, NMRI pc. vs. aqIGT pc.: 2*p* < 0.001, preIGT pc. vs. aqIGT pc.: 3*p* < 0.0001, NMRI d14.5 vs. aqIGT d14.5: 4*p* < 0.0001, NMRI pc. vs. aqIGT pc.: 5*p* < 0.0001, preIGT pc. vs. aqIGT pc.: 6*p* < 0.01, NMRI pc. vs. preIGT pc.: 7*p* < 0.05

Next, we examined body weight gain during gestation. Preconceptionally, preIGT and NMRI control mice showed comparable body weights, and both were significantly heavier than their aqIGT counterparts (Fig. 5A). As expected, at d14.5, body weights in all strains increased significantly with highest weights in NMRI controls (preIGT vs. aqIGT vs. NMRI: 36.0 ± 0.9 vs. 29.7 ± 0.8 vs. 46.2 ± 1.3 g; preIGT vs. NMRI: *p* < 0.01; preIGT vs. aqIGT: *p* < 0.05). The animals showed the highest body weights at d14.5 due to larger litter size. Albeit, no fetal macrosomia was detected (data not shown). In turn, the moderate increased weight of the preIGT model is due to a higher gonadal fat percentage compared to the NMRI and aqIGT strains (data not shown). The liver weights were measured at preconception and d14.5 to assess the hepatic weight response towards gestation. Preconceptionally, liver weights in the NMRI controls and preIGT mice were significantly higher than in the aqIGT strain (Fig. 5B). During gestation, all three strains showed increased liver weights to meet the metabolic demands of fetal development. Hence, the liver-to-body weight ratio was the lowest in the preIGT model, even stagnating compared to the other two strains (Fig. 5C). Both the pregnancy-related aqIGT and NMRI livers exhibited a robust elevation of the weight ratio compared to non-pregnant mice. In contrast, there was no liver-to-body weight ratio elevation in preIGT mice at d14.5 of gestation at all (preIGT pc. vs. preIGT: 5.29 ± 0.17 vs. 5.39 ± 0.22%) (Fig. 5C). The NMRI controls showed a significantly higher weight gain than the two comparators. Interestingly, the relative mass increase from preconception to gestation was significantly inferior in the preIGT mice compared to the comparators (preIGT vs. aqIGT vs. NMRI: 23.58 vs. 61.92 vs. 69.01%) (Fig. 5D). These data show that hepatic weight adjustment follows maternal body weight adjustment, whereby only an insufficient adaptation occurs in the preIGT model.

### Effects of gestation on lipid classes in the liver

**Fig. 6:**
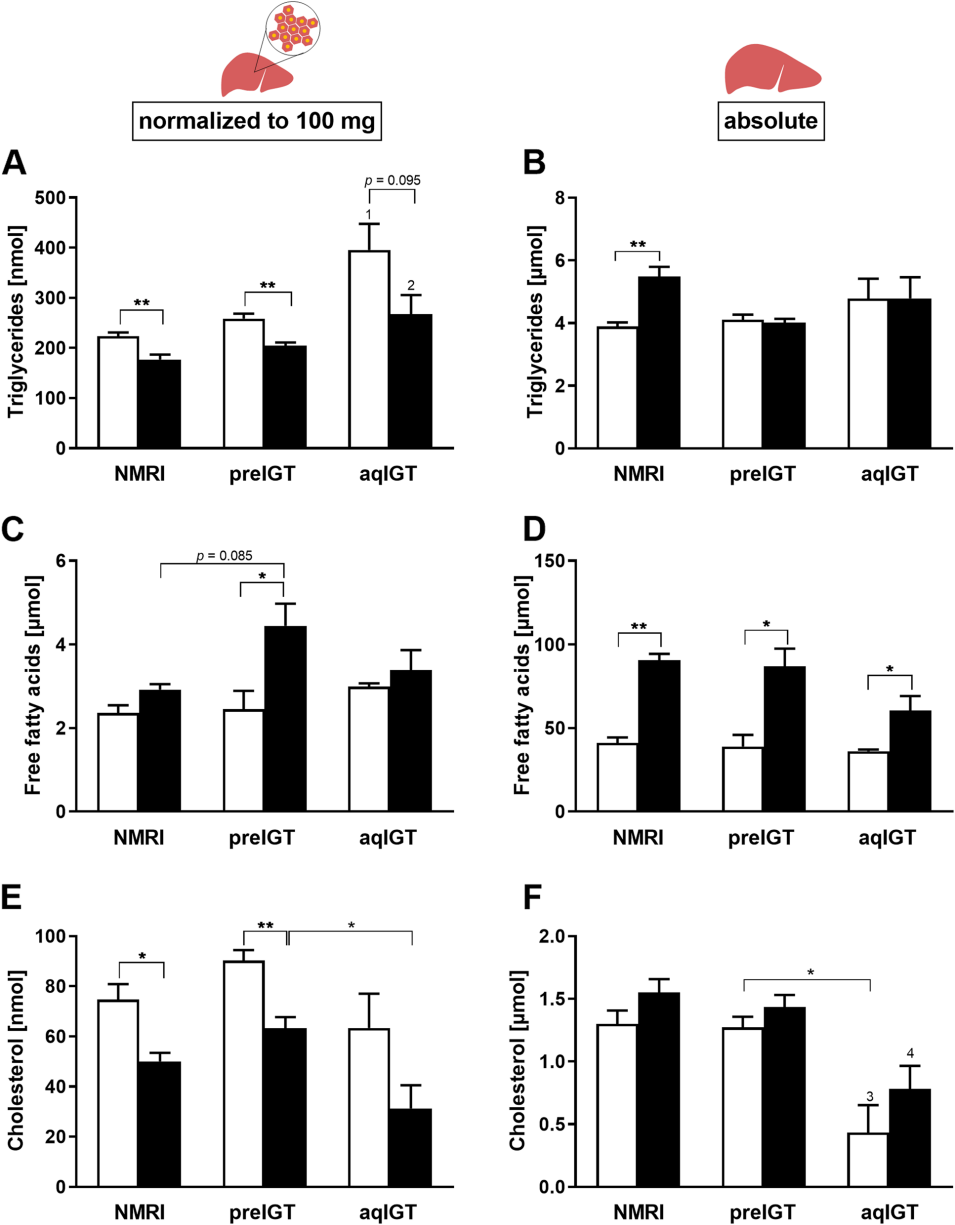
Impact of gestation on hepatic lipid classes. Relative (normalized to 100 mg hepatic tissue) and absolute (normalized to whole liver weight) **A, B** triglyceride, **C, D** free fatty acids, and **E, F** cholesterol of mice with preexisting impaired glucose tolerance (preIGT), impaired glucose tolerance acquired during gestation (aqIGT), and NMRI mice, at time points preconceptional (pc.; white bars) and day 14.5 of gestation (d14.5; black bars). Data are presented as means ± SEM (**A, B, E** and **F** *n* = 5–6, **C** and **D** *n* = 3–5 animals per group). *p < 0.05, **p < 0.01, NMRI pc. vs. B6 pc.: 1*p* < 0.01, NMRI d14.5 vs aqIGT d14.5: 2*p* < 0.05, NMRI pc. vs. aqIGT pc.: 3*p* < 0.05, NMRI d14.5 vs aqIGT d14.5: 4*p* < 0.05

Due to the different hepatic weight adjustments during gestation in the three strains (Fig. 5), we calculated both relative (normalized to 1 mg tissue) and absolute (normalized to absolute liver weight) changes in lipid concentrations. Interestingly, the elevated plasma lipids in preIGT mice were not associated with excessive lipid accumulation in the liver. Similar ratios between preconceptional and pregnant animals were found in the relative calculation for all strains studied, but to different extents depending on the considered lipid fraction. Thereby, all strains showed decreasing TG concentrations during gestation (Fig. 6A), whereas absolute liver TG remained unchanged in both preIGT and aqIGT mice between the preconceptional and gestational state, but increased significantly in the NMRI controls (Fig. 6B). In contrast, the relative FFA concentrations in all strains developed in the opposite direction. Trends towards increased FFA concentrations were visible in all three strains, but only preIGT mice showed a significant increase at d14.5 (preIGT pc. vs. preIGT d14.5: 2.45 ± 0.44 vs. 4.44 ± 0.53 µmol; *p* < 0.05) (Fig. 6C). The absolute FFA concentrations showed the same tendency, although the gestation-related increase was more pronounced and significant in all strains (Fig. 6D). Furthermore, all strains showed decreasing CL concentrations during gestation according to the relative calculation (Fig. 6E). In contrast, absolute CL showed an increase, with preconceptional preIGT mice and NMRI controls exhibiting significantly higher concentrations compared with aqIGT mice at both time points (Fig. 6F). A noticeable shift within the sphingolipid fractions was observed. AqIGT mice and NMRI controls displayed a significant increase in SM in relative and whole liver calculations, at both time points, preconception and d14.5.

**Fig. 7:**
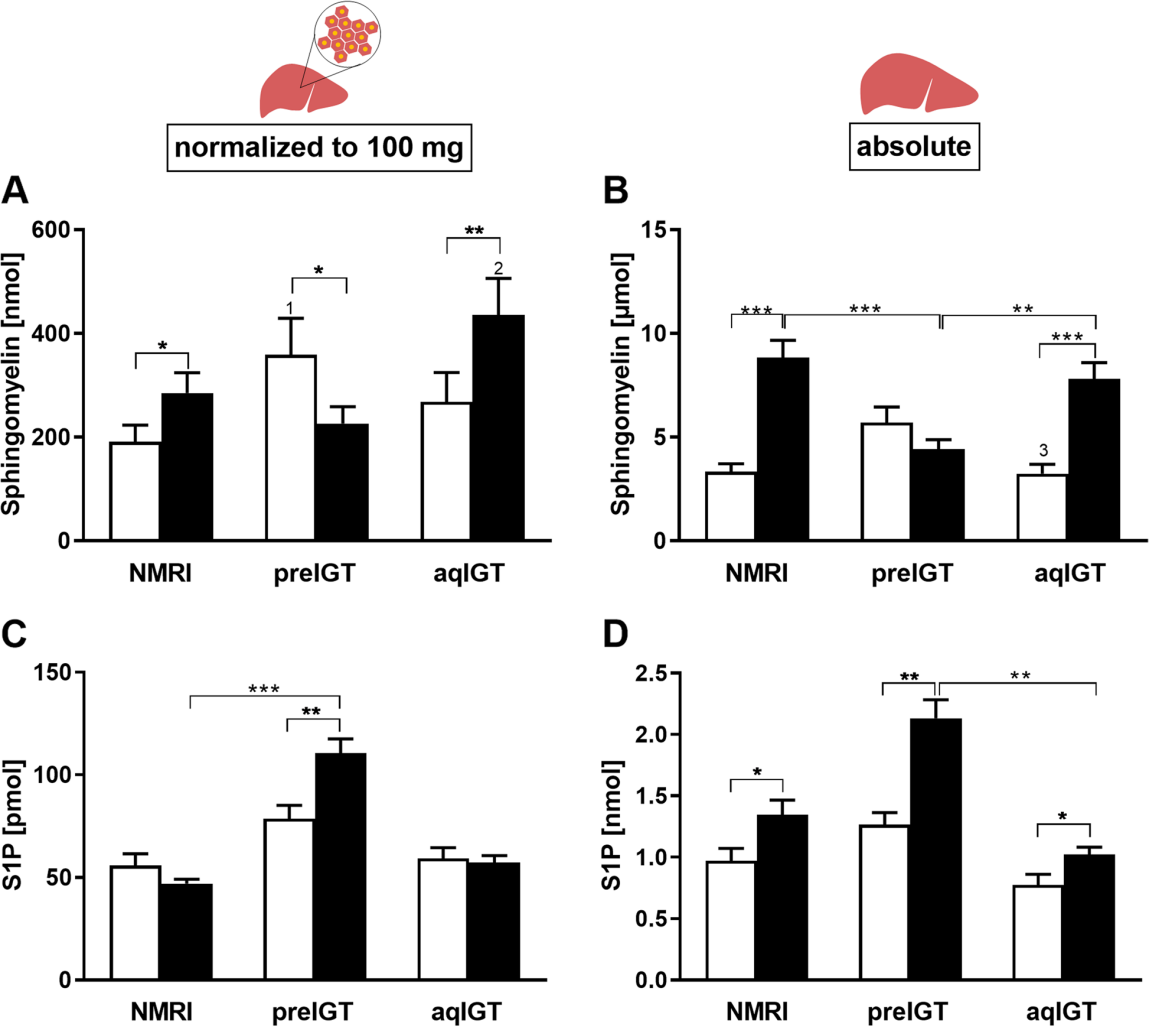
Opposite shifts in the sphingolipids during gestation. Relative (normalized to 100 mg hepatic tissue) and absolute (normalized to whole liver weight) **A, B** sphingomyelin and **C, D** sphingosine-1-phosphate (S1P) of mice with preexisting impaired glucose tolerance (preIGT), impaired glucose tolerance acquired during gestation (aqIGT), and NMRI mice, at time points preconceptional (pc.; white bars) and day 14.5 of gestation (d14.5; black bars). Data are presented as means ± SEM (**A** and **B** *n* = 12–15, **C** and **D** *n* = 6 animals per group). *p < 0.05, **p < 0.01, ***p < 0.001, NMRI pc. vs preIGT pc.: 1*p* < 0.05; preIGT d14.5 vs. aqIGT d14.5: 2*p* < 0.001; preIGT pc. vs. aqIGT pc.: 3*p* < 0.05

In contrast, in the preIGT mice, SM decreased significantly during gestation, both relatively and absolutely (Fig. 7A,B). At d14.5, both the aqIGT and NMRI controls showed significantly higher SM than the preIGT mice (preIGT vs. aqIGT vs. NMRI: 4.43 ± 0.45 vs. 8.84 ± 0.83 vs. 7.80 ± 0.79 µmol; preIGT vs. NMRI: *p* < 0.001, preIGT vs. aqIGT: *p* < 0.01). In contrast, relative S1P concentrations were already slightly increased preconceptionally in preIGT mice and significantly increased at d14.5 compared to NMRI controls (preIGT vs. aqIGT vs. NMRI: 42.01 ± 2.59 vs. 21.74 ± 1.27 vs. 17.85 ± 0.81 nmol; preIGT vs NMRI: p < 0.001, preIGT vs. aqIGT: p = 0.080). Both aqIGT mice and NMRI controls remained unchanged, equally leveled in their S1P concentrations (Fig. 7C). In the absolute calculation, all three strains showed a significant increase during gestation. However, the preIGT animals exhibited significantly higher S1P than the aqIGT mice and even slightly higher S1P than the NMRI controls (Fig. 7D).

### Absent CD36 translocation in preIGT mice

**Fig. 8:**
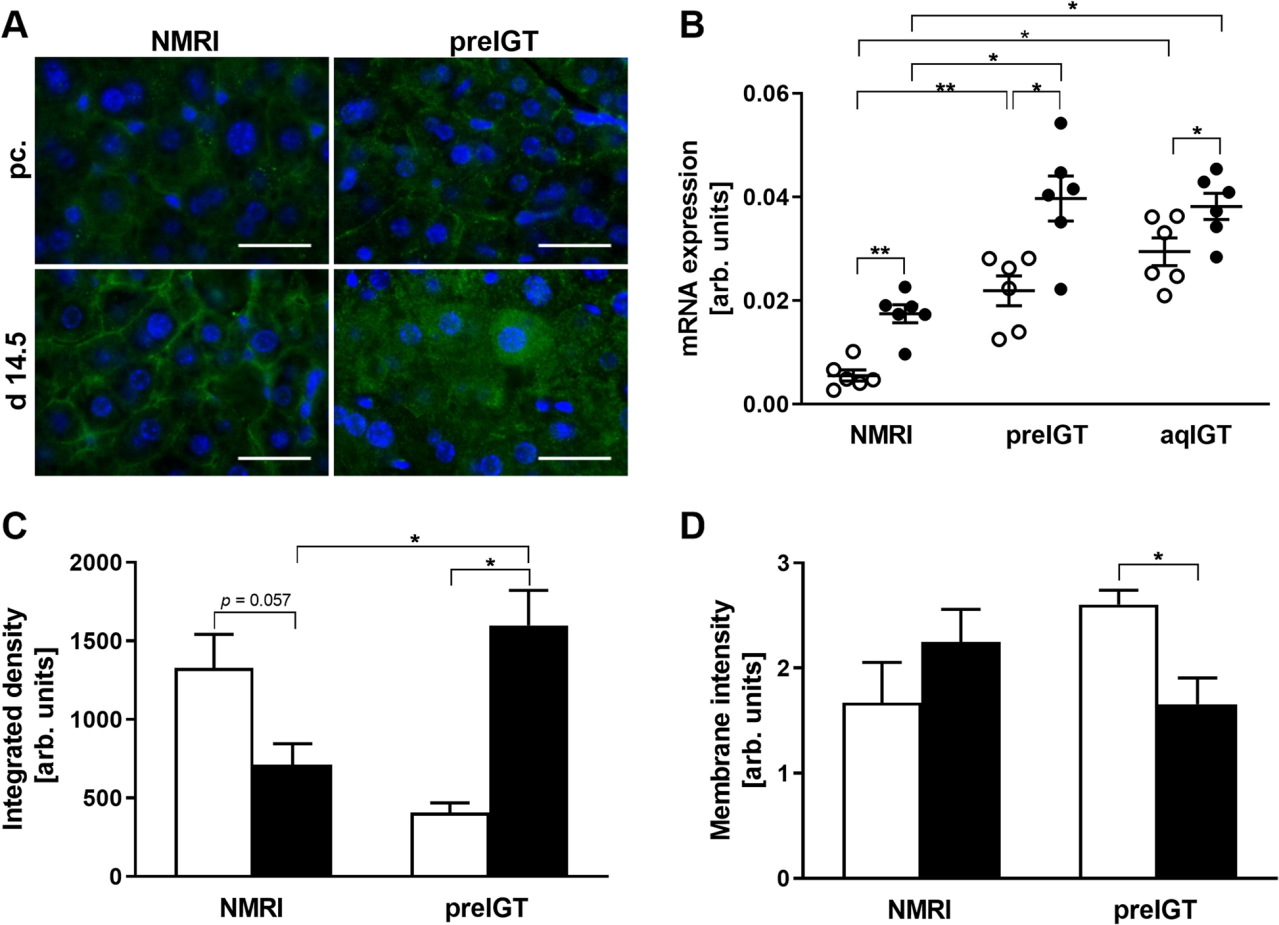
Dysfunctional fatty acid transport in pregnant preIGT mice. **A** Representative immunofluorescence staining for CD36 (green) in hepatocytes of mice with preexisting impaired glucose tolerance (preIGT), and NMRI mice at time points preconceptional (pc.) and day 14.5 of gestation (d14.5). Nuclei were stained with DAPI (blue). Scale bars = 25 μm. **B** Hepatic *Cd36* gene expression of preIGT mice, mice with impaired glucose tolerance acquired during gestation (aqIGT), and NMRI mice at time points pc. (open circles) and d14.5 (solid circles). **C** Quantification of integrated immunofluorescence density. **D** Quantification of membrane located immunofluorescence (IF) of CD36 stained liver sections of preIGT and NMRI control mice at time points pc. (white bars) and d14.5 (black bars). Data are presented as means ± SEM (**B** *n* = 6, **C** and **D** *n* = 3–5 animals per group) in arbitrary units. *p < 0.05, **p<0.01

To investigate possible functional mechanisms for the alterations in lipid metabolism, fatty acid transport protein CD36 was examined (Fig. 8A-D). In preIGT mice, as well as in both other strains, hepatic *Cd36* gene expression was significantly increased from preconception to d14.5 (Fig. 8B). Compared to NMRI controls, preIGT and aqIGT mice revealed significantly higher *Cd36* expression levels at both times, before and during gestation. For further characterization, the protein levels in the preIGT mice were examined in comparison to NMRI controls (Fig. 8A). According to the mRNA-expression levels, pregnant animals in both strains showed increased immunofluorescence. However, the integrated fluorescence density exhibited contrary patterns when comparing preconception and d14.5 both strains (Fig. 8C).

**Fig. 9:**
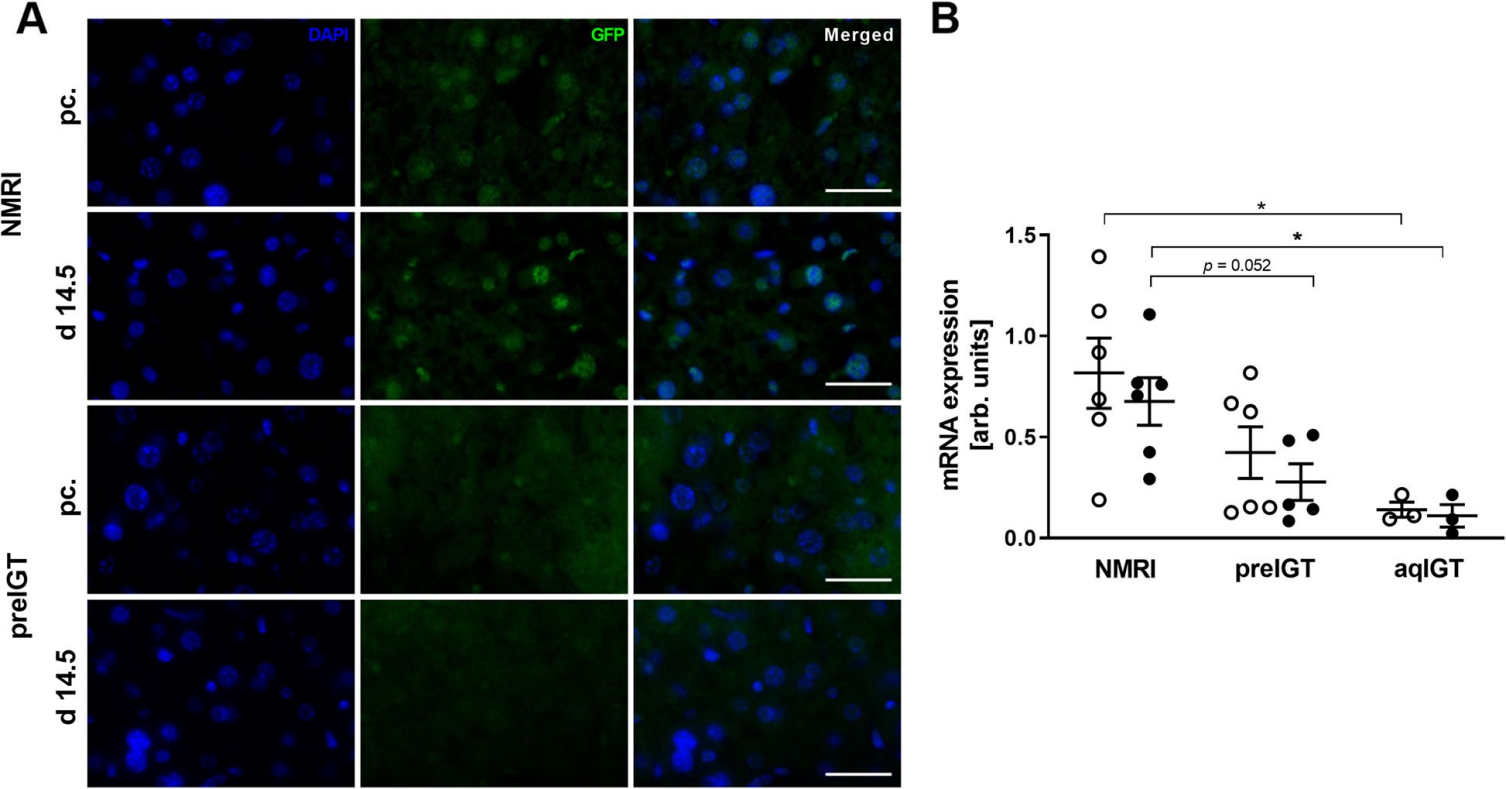
Less hepatic PPARα expression in preIGT mice. **A** Representative immunofluorescence staining for PPARα (green) in hepatocytes of mice with preexisting impaired glucose tolerance (preIGT), and NMRI mice at time points preconceptional (pc.) and day 14.5 of gestation (d14.5**)**. Nuclei were stained with DAPI (blue). Scale bars = 25 μm. **B** Hepatic *Pparα* gene expression of preIGT mice, mice with impaired glucose tolerance acquired during gestation (aqIGT), and NMRI mice at time points pc. (open circles) and d14.5 (solid circles). Data are presented as means ± SEM (**B ***n* = 3–6 animals per group) in arbitrary units. *p< 0.05

Thus, the preconceptional integrated density was decreased in the preIGT mice compared to the controls. Further, the preIGT livers showed a significant, almost fourfold increase at d14.5 compared to preconception, while the density in the NMRI controls markedly decreased (preIGT vs. NMRI: 1599.82 ± 222.47 vs. 711.83 ± 134.59; p < 0.05). Consequently, the cellular distribution measured as hepatocellular CD36 membrane intensity was determined to be opposite. There was a significant decrease in the preIGT mice at d14.5, while no significant changes were observed within the NMRI controls, implying insufficient translocation to the plasma membrane of hepatocytes in preIGT mice (Fig. 8A,D). Membrane-associated FATPs, like CD36, are regulated by PPARs, a group of nuclear receptor proteins that function as transcription factors. Therefore, PPARα was investigated in preIGT mice and NMRI controls (Fig. 9A,B).

Gene expression was lower in the preIGT and aqIGT mice at both time points. *Pparα* was significantly more abundant in the NMRI controls preconceptionally and at d14.5 than in the aqIGT animals. Compared to the preIGT mice, the controls also showed markedly increased mRNA expression at d14.5 (Fig. 9B). To verify the gene expression values, protein expression were investigated by immunofluorescence staining in preIGT and NMRI mice. While the fluorescence intensity between non-pregnant preIGT and NMRI controls did not differ, the fluorescence in the preIGT mice decreased at d14.5.

## Discussion

In the present work, we examined two mouse models that differ in terms of the onset of IGT in relation to gestation, although both are considered models of GDM. The C57BL/6 N strain, referred to as the aqIGT model, shows the classical clinical manifestation. Thus, the phenotype appears first during gestation. In contrast, the NZO mouse, referred to as the preIGT model, already exhibits IGT preconceptionally. If examined by OGTT during pregnancy, as is done in pregnant humans, the two models could not be distinguished at the time of diagnosis in terms of IGT. With this study, we were able to show that the different onset of IGT was associated with significant differences in important metabolic parameters. These include differences in S1P, FFA, and TG concentrations in plasma as well as differences in hepatic SM, adaptive liver weight gain, and differences in the expression and/or cellular localization of CD36 and PPARα.

Glucose tolerance is even impaired in normal pregnancy up to a certain degree, due to physiological adaptation of maternal metabolism to the higher metabolic demand of fetal development. The C57BL/6 N strain represents a model of diet-induced obesity, but it has been shown that the strain is generally susceptible to IGT, similar to the C57BL/6 J substrain. The substrains differ in the form of nicotinamide nucleotide transhydrogenase (NNT), which is usually considered the main cause of IGT. However, data show that C57BL/6 N as a wild-type strain has similar GSIS and glucose tolerance compared to the C57BL/6 J strain [60–62]. Interestingly, female mice already showed IGT under SD during gestation. Therefore, our data revealed that the C57BL/6 N strain, despite being commonly used as control strain, exhibits GDM model characteristics too. The preIGT model showed no hyperglycemia during the study, but already a preconceptional glucose tolerance disorder. There is evidence for adverse pregnancy outcomes associated with preconceptional IGT independent of obesity as indicated by BMI [63]. With a glucose tolerance disorder already present before conception that endures during pregnancy, the preIGT model represents a wider range of disorders than the strict definition of GDM. The improvement of insulin secretion in the preIGT model during gestation indicated by a better stimulability of insulin secretion but lower insulin concentrations in the OGTT may be confusing at first glance but was attributed to extrapancreatic effects, such as protective estradiol or cytokines [19, 64, 65]. In fact GDM subtypes with insulin secretion disorders without impaired insulin sensitivity have already been stratified [66]. This indicates the bright heterogeneity of the disease and shows the need for appropriate models for its complexity.

Plasma FFA concentrations were the most prominent differences in measured lipid fractions between subtypes during pregnancy, with notably increased concentrations in the preIGT model. FFA can be important mediators of peripheral IR and have a tendency to be increased in women suffering from GDM [26]. From plasma FFA, a worsening of the metabolic situation cannot be derived for the aqIGT phenotype. A trend of higher values before the onset of IGT was noticeable even before conception compared to the controls and it was shown that changes in the measured plasma classes are already present in the prediabetic state [67]. Obese women showed a tendency for slightly higher FFA concentrations in subjects with future GDM even before conception, ongoing with a significant decrease in FFA suppression by insulin stimulation [68]. Thus, FFA concentrations are suitable as a marker for a specific preIGT subtype. Decreasing plasma insulin concentrations during gestation could therefore explain the markedly increased FFA due to enhanced lipolysis in the preIGT model. During gestation, the preIGT model also showed the highest increment in the other major plasma lipid fractions, as increased TG concentrations were associated with this phenotype. Although more associated with cardiovascular risk, the atherogenic index was significantly increased in the preIGT model during gestation. However, it is argued that this ratio is suitable for selecting patients who need an earlier and aggressive treatment of lipid abnormalities and can be used as a marker for IR [69, 70]. Additionally, as a potential risk factor, the index correlates positively with GDM [71]. The increase in plasma S1P has recently been discussed as a predictor of both, human and rodent obesity [46]. Although S1P concentration increased in all strains, they were significantly higher in the preIGT model. In the NZO mouse, elevated S1P concentration was observed after HFD and its inhibitory effect on insulin signaling could be reversed by an S1P2 receptor antagonist. Furthermore, microarray data showed increased gene expression in S1P signaling [31, 72]. This indicates a potential S1P-mediated increase in IR. When comparing sphingolipids during gestation, we observed a shift in distribution from the liver to plasma. The preIGT model showed unchanged plasma SM but increased plasma S1P during gestation, while the aqIGT model showed decreasing plasma SM. Contrary, the preIGT model showed decreasing hepatic SM abundance, while the aqIGT model showed a significant increase. Correspondingly, the S1P abundance behaved oppositely. There is evidence that sphingosine accumulates in primary rat hepatocytes after stimulation with palmitic acid, concomitant with an enhanced cellular export [73]. Such an extracellular transport in hepatocytes could be an important mechanism of protection against lipotoxicity and a possible explanation for the high plasma sphingolipid concentrations of the preIGT model. Hence, the SM fraction functions as a reservoir for sphingolipid turnover and rapid generation of its precursors [74, 75]. Given the decreasing hepatic SM reservoir of the preIGT model, we assumed a shift towards ceramide fraction by SM hydrolysis. This was supported by the observed increase in hepatic S1P, a metabolite of ceramide metabolism. Elevated S1P is a characteristic of the preIGT model in both compartments and is a marker of impaired lipid metabolisms in diabetic rodent models [31, 76]. Fittingly, diabetic Ins2Akita mice were shown to have reduced hepatic SM [76].

To clarify alterations in other lipid fractions depending on the preconceptional or pregnant state, the assessed fractions were also examined in the liver. In pregnant women, MRI analyses have already shown that beyond body size, a higher metabolic demand is responsible for the enlargement of the liver. Consistent with rodent data indicating that liver size remains elevated through lactation even though body size is reduced compared to late pregnancy [77]. Interestingly, the preIGT mouse revealed altered hepatic weight adaptation during gestation and even a reduced liver-to-body weight ratio. The increase in body weight during gestation was also significantly lower in the preIGT model compared to control animals. It has already been shown that weight gain in women with GDM is lower than in metabolically healthy pregnant controls, but this might be due to higher pregravid weights [78]. Because of the lacking adaptation and to account for a striking reduced percentage of hepatic weight gain in the preIGT, liver lipid fractions were determined both, relatively and absolutely, as it reflects the total lipids of the functioning organ, which is useful for considering the plasma-liver relationship under in vivo conditions.

All models, including the NMRI control strain, showed stagnant to decreasing relative lipid concentrations during gestation. Thus, no significant differences beyond a pregnancy-related effect in the specific lipid fractions were evident, apart from the characteristic differences in the sphingolipid fraction. Because of the severity of the invasive procedure and due to the limited availability of suitable imaging techniques, data on hepatic lipids in pregnant women are limited; few data are available from preeclamptic women who exhibited fatty infiltration [79, 80]. Data for rodent models are also scarce, though if available they are from nutritional intervention studies, often on HFD, which complicates a comparison of pregnancy-induced effects on SD. Several studies revealed indifferent data, with a dynamic state of lipids during pregnancy, temporal changes in hepatic lipids, increments in TG and CL in early pregnancy, and unchanged FFA. Later in pregnancy, hepatic TG and CL returned to preconceptional proportions, suggesting a reduced hepatic de novo lipogenesis [81, 82]. Contrary, further rodent studies revealed unchanged or even decreased hepatic TG during gestation [83, 84]. The condition of decreased relative liver lipid values seems to be attributable to pregnancy since fetal energy needs must be met in addition to maternal metabolism. The absolute increase, in turn, related to the total liver weight, showed very illustratively that pregnancy is an enormously demanding condition. In relation to only a small increase in lipids, an enormous increase in organ mass is required. Since the preIGT model showed the same trends in hepatic lipid concentrations as the aqIGT model and did not exhibit TG accumulation, the high plasma concentrations are a consequence of low hepatic adaptation. In this case, a lower, possibly saturated storage capacity of the preIGT liver leads to higher lipid concentrations in plasma to maintain hepatic lipid balance.

Consequently, due to the remarkably elevated plasma FFA concentrations of the preIGT model during gestation, the FFA functional metabolic pathway containing PPARα and CD36 was investigated. CD36 has already been shown to be a valuable marker in diabetic NZO mice [17]. CD36, necessary for the uptake of FFA into the hepatocytes, is associated with both glucose and lipid metabolism, thereby establishing it as a susceptible marker for metabolic abnormalities and adaptation defects [85, 86]. JAK2L mice with increased FFA concentrations show a significantly increased expression of hepatic CD36 and fatty liver disease [87]. However, mice with CD36 deletion exhibit improved whole-body insulin sensitivity [87]. *Cd36* gene expression was elevated in both the preIGT and aqIGT model, as well as the control, proving that pregnancy induces *Cd36*. This is a consequence of a state of enormous energy demand and is supported by data showing an elevation of *Cd36* mRNA in mice until day 19 of gestation [88]. However, both the preIGT and the aqIGT model revealed overall higher *Cd36* expression patterns compared to controls. This is in line with findings demonstrating increased *Cd36* expression in non-alcoholic fatty liver disease patients as well as contributing significantly to dyslipidemia in mice [89, 90]. Therefore, *Cd36* expression also seems to be a marker for metabolic deterioration during pregnancy. Interestingly, the preIGT model revealed a different pattern of hepatocytic protein expression during gestation, with cytosolic rather than membranous localization compared to the NMRI controls. For the aqIGT model, CD36 localization at the plasma membrane, as in the NMRI control, could already be shown to be analogous to the physiological adaption of pregnancy on HFD [91]. In isolated hepatocytes from obese Zucker rats, elevated membrane-bound CD36 expression were not simply caused by increased cellular CD36 expression but were insulin-induced, and even higher insulin concentrations were required for CD36 translocation compared to lean controls [92]. This demonstrates the insulin dependence of FFA transport, as translocation enables to response on the prevailing metabolic state. The lack of translocation in the preIGT model is thus an expression of insufficient insulin stimulation caused by relatively reduced insulin secretion with lower insulin concentrations. Hepatic insulin responsiveness is dependent on sphingolipids as well. C2-ceramide was administered to rat pubs, which developed hyperglycemia and hyperlipidemia as adolescents accompanied by increased serum ceramides [27]. The livers of these treated rats further showed reduced lipids but increased ceramide concentrationen. It could thus be shown that a derivative of S1P has the ability to counteract insulin signaling. It is reasonable to assume that the characteristically elevated S1P concentrations of the preIGT model may also contribute to the lack of CD36 translocation during gestation. Furthermore, the correct functionality of CD36 depends on co-polymerization with caveolin-1 in SM-rich membrane microdomains, the so-called caveolae or lipid rafts [93, 94]. Thus, reduced SM in the liver of the pregnant preIGT model, both relatively and absolutely could be another reason for the lack of CD36 translocation. This implies that sphingolipids also emerge as an important marker fraction for altered metabolism and should be considered for the missing CD36 functionality in the preIGT model.

*Pparα*, mainly expressed in the liver due to its high FFA turnover rate, is a transcriptional regulator of the *Cd36* gene. At first glance, this seems to contradict our data, as we observed decreasing *Pparα* with simultaneous increased *Cd36* gene expression. However, *Cd36* expression is also induced by PPARγ and other transcription factors such as the pregnane X receptor (PXR), which might explain our observation [86]. Further, estrogen potentially inhibits the beneficial effects of PPARα on obesity and lipid metabolism through its effects on PPARα-dependent gene regulation [95, 96]. This inhibitory capacity could be important in the state of pregnancy with rising estrogen concentrations:. In addition, decreased mRNA expression of *Pparα* was also observed in streptomycin-induced diabetic rats, as well as in Akita and *db/db* mice. Polymorphisms in T2DM patients could also be identified which led to altered PPARα function accompanied by altered plasma lipid concentrations [97, 98]. This demonstrates the link between PPARα and dyslipidemia in diabetes. Therefore, the observed downregulation in the pregnant preIGT model may be a distinct marker gene for this prediabetic phenotype. However, the activation of PPARα also increases hepatic gene expression of serine-palmitoyltransferase (SPT) in mice, which is involved in sphingolipid de novo synthesis. Further, PPARα regulates the gene expression of long-chain acyl-CoA synthetase. Although the data are limited, it shows that the initial step of sphingolipid synthesis, involving palmitoyl-CoA and SPT, is regulated to a certain extent by PPARα, mediating gene expression and enzyme regulation [99, 100]. The direct interaction between S1P and PPARγ has already been reported in human endothelial cells, suggesting it is a possible interaction partner of PPARα as well. Interestingly, the interaction results in suppression of *Cd36* gene expression, although this was not seen in response to S1P treatment at the protein level [101].

Although revealing interesting insights into the lipid metabolism of different GDM subtype models, it should be mentioned that our data have limitations. For the measurement of lipid classes in the liver, we used a densitometric TLC method, which is an established, fast and highly versatile separation technique, but a method with low resolution. In further studies, this method should be extended by mass spectrometric analyses. We could not measure ceramides or sulfatides, sphingolipid fractions that are modulated by PPARα and appear to be associated with diabetes [102]. Additionally, the preIGT phenotype could be a consequence of altered sensitivities to hormone stimuli in pregnancy, as well as epigenetic alterations in DNA methylation patterns, which should be further assessed [103–105]. It is also important to point out interspecies differences, as mice generally have tenfold higher hepatic *Pparα* gene expression than humans [106, 107]. Onwards, as a transcription factor, the transcriptional activity of PPARα is more important than its mere gene or protein expression. However, we could not determine its functional expression in this study.

Of note is the use of the C57BL/6 N strain as a model for aqIGT, whereas it is often used as a control in metabolic studies. An explanation is that the mice developed marked IGT during gestation in OGTTs with an oral glucose bolus of 2 mg glucose/g body weight by gavage to foster robust differences in blood glucose and plasma insulin [44]. However, this was only evident when the NMRI strain was used as a control. In our opinion, the C57BL/6 N strain is therefore not an appropriate control for diabetes studies.

## Conclusion

In summary, the different onset of IGT in the investigated models is accompanied by strong differences in lipid metabolism. A decrease in preconceptionally elevated insulin concentrations during gestation in the preIGT model resulted in a downregulation of insulin signaling. This resulted in a lack of CD36 translocation to the plasma membrane, reducing hepatic FFA uptake and increasing plasma FFA concentrations. Furthermore, we have demonstrated a decrease in the hepatic SM reservoir associated with increased plasma S1P concentrations, a known marker of impaired lipid metabolism in diabetic rodent models, thus exerting an inhibitory effect on insulin signaling. Presumably due to compensation of hepatic IR by increased GSIS, the preIGT model shows no deterioration of the overall metabolic state during gestation. However, since this model is known to develop manifest diabetes under metabolic stress conditions in later life, altered sphingomyelin metabolism during gestation could serve as an important marker, if not signal mediator, for higher risk of these later maternal metabolic complications. The latter, nevertheless, requires further investigation. However, characterization of models with different initiation periods of IGT has clearly shown that the classification of GDM into subcategories reveals different mechanisms of pathogenesis and thus different possibilities for intervention.

## Data Availability

The datasets used and analyzed during the current study are available from the corresponding author on reasonable request.
